# Diseases of the Nervous System

**Published:** 1894-12-01

**Authors:** 


### DISEASES OF THE NERVOUS SYSTEM.

**Syringomyelia.**—Now that this disease is more generally recognised, cases of it are being recorded with considerable frequency. Mr. Lunn,[1] at the Clinical Society, recorded a case which he had watched for over ten years, and upon which he finally obtained a post-mortem examination. Besides the cardinal sensory symptoms there had been present during life muscular wasting and trophic changes in the nails, skin, and several joints of the upper extremities. Dilatation of the central canal of the spinal cord in the cervical and upper dorsal regions had led to destruction of ganglion cells. The dislocated joints were typical examples of "osteo-arthritis." Marie,[2] at the Medical Society of the Hospitals, showed a case in which the right hand and left foot resembled those in acromegaly, the hypertrophy in the hand being especially marked at the heads of the metacarpal bones and the fingers. Hoffman has given this condition the better name of *cheiromegaly*, to avoid confusion with acromegaly proper, with which there is essentially nothing in common. Singer,[3] of Vienna, showed a case of the type of Morvan's disease. Bullæ on the arm and forearm had been followed by cicatrices; paronychia of the thumb had led to necrosis of the distal phalanx; the fingers were thickened. A diagnosis of leprosy was unhesitatingly rejected, as the patient had never been in a leperous country. C. K. Mills,[4] of Philadelphia, records two cases. One of these cases is specially interesting, inasmuch as the symptoms were strictly unilateral. Unlike the majority of cases the morbid phenomena were more developed in the lower extremity than in the upper, where, however, fibrillary tremor existed in the muscles. There was much wasting and partial paralysis of the muscles of the right thigh, and in the course of four years the knee had become greatly swollen, and changed in shape. When the patient was standing or walking the limb presented the appearance of an arc of a circle with the convexity backward. F. X. Dercum[5] brought before the Philadelphia Neurological Society a case in which an autopsy had been obtained. No trophic lesions besides muscular wasting are mentioned. A large cavity was found occupying the gray matter of the spinal cord, most marked in the cervical, and to a less extent in the dorsal region. The anterior cornua appeared as though lopped off, and posteriorly the cavity encroached so much that the posterior roots and root-zones could hardly be defined. In the discussion upon this case, Charles W. Burr said he had made an autopsy upon a case of syringomyelia, in which there had been no sensory changes whatever. Dercum, in reply, said he thought that in the joint changes we may have a point of differential diagnosis between chronic polio-myelitis and syringomyelia. Professor Sonnenburg[6] records a case of syringomyelia in which great arthropathy existed in one shoulder-joint and the finger joints were enlarged. It is evident that arthropathy is considerably more frequent in syringomyelia than in locomotor ataxy.

**Spinal Arthropathy.**—W. B. Noyes,[7] in commenting upon the nature of "Charcot Joint," remarks that the condition is simulated by arthritis deformans, in which there is also a chronic history, affecting a series of joints, especially the smaller ones. It is rare to get the abnormal laxity of joints so characteristic of arthropathica tabica, but rather abnormal stiffness. Effusion, swelling, œdema, all appear late, if at all, in arthritis deformans. The pain is sometimes very severe, while in the Charcot joint the absence of pain is always a marked feature. Jürgens and Marie have shown that when no arthritis such as could be diagnosed during life existed, still alterations in the ligaments and bony structures of the great joints have been found post-mortem. Charcot joints have been found also many in times the pre-ataxic stage of tabes. Weir-Mitchell quotes several cases of joint disease following wounds and injuries to the nerves; and Hausemann[8] also gives a case of arthropathy following injury to the sciatic nerve. Noyes thinks upon the whole that possibly the peripheral nerves determine joint changes more than the cord itself. James Hendrie Lloyd at Philadelphia Neurological Society (5) showed a case of progressive muscular atrophy with arthropathies of the knee joints. The case presented the muscular, motor, atrophic, joint and skeletal changes of syringomyelia, but not the sensory phenomena.

Prantois and Étienne[9] relate a case of advanced progressive muscular atrophy of some years standing. On the right side the head of the humerus was dislocated upwards, and grating was caused upon movement of the bone. There was also bi-lateral carpo-radial subluxation, and the outer end of the left clavicle was mobile and dislocated; creaking could also be obtained in the left shoulder joint.

**Ataxy.**—Déjérine,[10] in the laboratory reports of M. Vulpian, reports two cases in which during life there was marked ataxy and absent knee-jerks; in the "Semaine Medicale" he quotes another similar case. In each of these cases the spinal cord was found normal post-mortem, but parenchymatous neuritis was discovered. *Per contra*, Dr. Ransom[11] brought before the Nottingham Medical Society the case of a woman who had not been ataxic during life, but in whose spinal cord after death extensive degeneration of the posterior roots and posterior columns was found.

**Non-hereditary Friedreich's Disease.**—Dr. Hector Mackenzie[12] records a perfectly typical case (but without speech defect) in a girl thirteen years of age. It had began after measles at seven years of age, and the family history showed entire freedom from tendency to nervous disorder.

**Tendon Reflexes.**—It is accepted now as a fact that in cases of complete transverse lesion of the spinal cord the knee-jerks are lost, and conversely it is stated by Charlton Bastian, Thorburn, and others that when a spinal cord lesion causes complete paralysis of motion and sensation in the legs the deep reflexes are always lost. Dr. E. Reynolds,[13] however, records two cases in which there was total loss of sensation and of power of motion in the lower limbs; in one the knee-jerks were markedly exaggerated, in the other they were absent, though the superficial reflexes were increased. Dr. P. Marie[14] calls attention to another tendon reflex, which he calls "contralateral." If the patellar tendon of one leg be struck when both feet are placed upon the ground a foot or so apart, the knee joint being bent at an obtuse angle, the adductor tendons of the other thigh contract, even though the knee-jerk be absent (owing to the disease of the spinal cord from the leg which is struck. This phenomenon, Marie thinks, is a proof that the knee-jerk is a true spinal reflex, and that it will serve as a test to distinguish those cases in which the loss of the knee-jerk is due to an alteration in the peripheral nerves from those in which it results from a lesion of spinal motor centres. [This reaction exists in many, but not in all normal persons, and is well marked in the spastic types of paraplegia; the limits of its diagnostic value are not yet determined.—*Abstractor.*]

[1] Med. Week, May 18, 1894. [2] Med. Week. April 13, 1894. [3] Med. Week, April 27, 1894. [4] Journ. Nerv. and Mental Dis., April, 1894. [5] Journ. Nerv. and Mental Dis., March, 1894. [6] Neurolog. Centralblat., quoted in Lancet, June 2, 1894. [7] Medical Record, June 16, 1894. [8] Berlin Klin. Woch., February, 1894. [9] Rev. de Med.. April, 1894, quoted in Ep. Brit. Med. Journ., March 26, 1894. [10] Quoted in Lancet, April 28, 1894. [11] Lancet, April 28. [12] Amer. Jour. Med. Sciences, April, 1894. [13] Med. Chron., June, 1894. [14] Med. Week, April 20, 1894.

